# Characteristics of Late-Onset Spondyloarthritis: Data from the Moroccan Registry of Biological Therapies in Rheumatic Diseases

**DOI:** 10.7759/cureus.39100

**Published:** 2023-05-16

**Authors:** Ahmed Mougui, Zineb Baba, Ihsane Hmamouchi, Redouane Abouqal, Ahmed Bezza, Fadoua Allali, Rachid Bahiri, Imad Ghozlani, Hasna Hassikou, Linda Ichchou, Saadia Janani, Taoufik Harzy, Redouane Niamane, Abdellah El Maghraoui, Imane El Bouchti

**Affiliations:** 1 Rheumatology, EzarriHospital, Faculty of Medicine and Pharmacy of Marrakech, Mohammed VI University Hospital, Marrakech, MAR; 2 Rheumatology, Arrazi Hospital, Faculty of Medicine and Pharmacy of Marrakech, Mohammed VI University Hospital, Marrakech, MAR; 3 Laboratory of Biostatistics, Clinical Research and Epidemiology, Mohammed V University, Rabat, MAR; 4 Laboratory of Biostatistics, Clinical Research and Epidemiology., Mohammed V University, Rabat, MAR; 5 Rheumatology, Military Hospital Mohammed V, Ibn Sina University Hospital, Rabat, MAR; 6 Rheumatology B, El Ayachi Hospital, Ibn Sina University Hospital, University Mohamed V, Rabat, MAR; 7 Rheumatology A, El Ayachi Hospital, Ibn Sina University Hospital, University Mohamed V, Rabat, MAR; 8 Rheumatology, Faculty of Medicine and Pharmacy, University Ibn Zohr, Agadir, MAR; 9 Rheumatology, Military Hospital Moulay Ismail, Hassan II University Hospital, Meknes, MAR; 10 Rheumatology, Mohammed VI University Hospital, Mohammed I University, Oujda, MAR; 11 Rheumatology, Ibn Rochd University Hospital, Casablanca, Morocco, Casablanca, MAR; 12 Rheumatology, Hassan II University Hospital, Fes, MAR; 13 Rheumatology, Military Hospital Avicenne, Mohammed VI University Hospital, Marrakech, MAR; 14 Rheumatology, Private Medical Office, Rabat, MAR; 15 Rheumatology, Ezarri Hospital, Faculty of Medicine and Pharmacy of Marrakech, Mohammed VI University Hospital, Marrakech, MAR

**Keywords:** diagnosis, characteristics, registry, late onset, spondyloarthritis

## Abstract

Introduction

The Assessment of SpondyloArthritis International Society (ASAS) criteria for axial and peripheral spondyloarthritis (SpA) allow for the classification of patients with an age of onset of disease of less than 45 years. However, SpA can start after this age. This study aimed to assess the characteristics of late-onset SpA (SpA>45 years) in the Moroccan registry of biological therapies in rheumatic diseases (RBSMR).

Methods

A cross-sectional study was conducted using the baseline data of the RBSMR. The protocol for the original RBSMR study was reviewed and approved by the Ethics Committee for Biomedical Research Mohammed V University - Rabat, Faculty of Medicine and Pharmacy of Rabat (approval number for the study was 958/09/19, and the date of approval was September 11, 2019), and all patients had given their written consent. Patients who met the 2009 ASAS criteria for SpA were included. They were divided into two groups: early-onset SpA (≤ 45 years) and late-onset SpA (>45 years). Clinical, biological, radiological, and therapy data of the two groups were compared. Statistical analysis was performed using SPSS v25 software (IBM Corp. Armonk, NY). Parameters with a p-value ≤0.05 were considered significant.

Results

Our population consisted of 194 patients. Thirty-one patients (16%) had late-onset SpA. Comparison between patients with early-onset (≤45 years) and late-onset SpA (>45 years) revealed that late-onset SpA had a higher tender joint count (p=0.01), a higher swollen joint count (p=0.02), depression (p=0.00), fibromyalgia (p=0.001), hypercholesterolemia (p=0.01), and a lower frequency of coxitis (p=0.008). Logistic regression analysis confirmed that late-onset SpA was associated with a higher tender joint count (OR=0.93, CI 95%: 0.88-0.98), a higher swollen joint count (OR=0.92, CI 95%: 0.85-0.99), depression ( OR=0.19, CI 95%:0.04-0.38), fibromyalgia (OR=1.75, CI 95%: 1.74-17.85), and a lower frequency of coxitis ( OR=0.29, CI 95%: 0.11-0.75).

Conclusion

As life expectancy increases, late-onset SpA will become increasingly common. It is therefore imperative to determine its characteristics. In the RBSMR study, late-onset SpA was associated with a high number of tender and swollen joints, depression, fibromyalgia, and a lower frequency of coxitis.

## Introduction

Spondyloarthritis (SpA) is a group of chronic inflammatory rheumatic diseases characterized by spinal enthesitis and sacroiliac joint involvement resulting in lower back and buttock pain, peripheral entheseal involvement mainly of the plantar fascia, which causes heel pain, peripheral joint involvement dominated by asymmetrical oligoarthritis of the large joints of the lower limbs, and specific extra-articular disorders such as psoriasis, IBD, and uveitis. It is also characterized by a particular genetic component, mainly HLA-B27, and a tendency to ankylosis [1,2]. It is most commonly found among young people and less frequently in the elderly [3]. The definition of late-onset SpA varies between teams from 35 to 55 years [4]. The ASAS criteria for axial and peripheral SpA classify patients with an age of onset of fewer than 45 years as SpA. However, SpA may begin after this age with a different phenotype than early-onset forms [5,6]. The aim of this study was to assess the clinical, biological, radiological, and therapeutic characteristics of late-onset SpA.

## Materials and methods

Author contributions

This article is a multicenter study conducted by the Moroccan Registry of Biotherapies (RBSMR), which involves the university hospitals of Marrakech, Rabat, Agadir, Oujda, Casablanca, Fès, and the military university hospitals of Agadir, Marrakech, Rabat, and Meknès. The authors of this article come from different centers and participated in the development of the registry, the definition of the methodology, and all work according to the criteria of the International Committee of Medical Journal Editors (ICMJE). They worked together to create the RBSMR, develop the methodology, collect the data, analyze the data, write the article, and ensure compliance with the ICMJE criteria.

RBSMR study

The Moroccan Registry of Biotherapies (RBSMR) is a multicentric historical-prospective registry of patients who were treated for rheumatoid arthritis (RA) or spondyloarthritis (SpA) with biologics in the rheumatology departments of university hospitals in Morocco. The inclusion period for the registry was from May 2017 to January 2019, and patients were followed up for three years. To be included in the registry, patients had to be over 18 years old and be treated for RA or SpA with biologic therapy (initiation or ongoing biotherapy). Patients, who were under biologic therapy for indications other than RA or SpA, as well as those with juvenile idiopathic arthritis, were excluded. A total of 440 patients were included in the registry, of which 419 were validated (225 with RA and 194 with SpA). The primary objective of the RBSMR registry was to assess the safety of biologics used in the management of RA or SpA. The secondary objectives were to evaluate the effectiveness of biologics in the real world and to assess the impact of biologics on the patient's quality of life. The protocol for the original RBSMR study was reviewed and approved by local institutional review boards and the national ethics committee, namely, the Ethics Committee for Biomedical Research Mohammed V University - Rabat, Faculty of Medicine and Pharmacy of Rabat. The approval number for the study was 958/09/19, and the date of approval was September 11, 2019. All personal data collected and analyzed were anonymized, and all patients had given their written consent.

Study designation and patients

This cross-sectional study was conducted using baseline data of the RBSMR and included 194 patients who fulfilled the ASAS criteria for axial SpA or peripheral SpA. Late-onset SpA was defined as SpA with an onset of symptoms greater than 45 years. Patients were divided into two groups: early-onset SpA (≤45 years) and late-onset SpA (45> years). The study compared various clinical and diagnostic characteristics, including age, gender, disease duration, family history of SpA, peripheral arthritis, inflammatory back pain, and enthesis tenderness. Radiological findings, such as X-ray, magnetic resonance imaging (MRI), and power Doppler ultrasonography, as well as biological characteristics like erythrocyte sedimentation rate (ESR), C-reactive protein (CRP), HLA-B27, and vitamin D levels, were also analyzed. Disease activity was assessed using several measures, including the Bath Ankylosing Spondylitis Disease Activity Index (BASDAI), Ankylosing Spondylitis Disease Activity Score-C-reactive protein (ASDAS-CRP), Bath Ankylosing Spondylitis Functional Index (BASFI), tender joint count, swollen joint count, and Visual Analogue Scale - Fatigue (VAS-F). Furthermore, the study evaluated comorbidities like depression, diabetes, fibromyalgia, latent tuberculosis, dyslipidemia, and osteoporosis, as well as extra-articular involvement in conditions like uveitis, psoriasis, and inflammatory bowel disease (IBD). Therapy data for both groups, including non-steroidal anti-inflammatory drugs (NSAIDs), analgesics, corticosteroids, and conventional synthetic disease-modifying antirheumatic drugs (csDMARDs) were analyzed.

Statistical analysis

Statistical analysis was performed using SPSS v25 software (IBM Corp., Armonk, NY). Quantitative variables were expressed as mean and standard deviation for variables with normal distribution or median and interquartile range when the distribution was not Gaussian. The normality of the variables was assessed by the Kolmogorov-Smirnov test. Categorical variables were expressed as numbers and percentages. Comparisons were made using the Chi² test for qualitative variables and the Mann-Whitney U or t-test for independent samples for quantitative variables depending on their distribution. Logistic regression was performed for variables with a p < 0.05.

## Results

Clinical and diagnostic characteristics of the population

The population consisted of 194 patients, of which 31 (16%) had late-onset SpA. Comparison between patients with early-onset (≤45 years) and late-onset (>45 years) disease did not show any significant differences regarding clinical and diagnostic characteristics (Table 1).

**Table 1:** Clinical and diagnostic characteristics of the population SpA, Spondyloarthritis

Parameters	Early-onset SpA (N=163)	Late-onset SpA (N=31)	p
Male N (%)	156 (95.7)	30 (96.7)	0.78
Disease duration (weeks)	636.97	503.07	0.09
Family history of SpA N (%)	21 (12.8)	5 (16.2)	0.78
Peripheral arthritis N (%)	111 (68.1)	23 (74.2)	0,50
Inflammatory back pain N (%)	156 (95.7)	30 (96.7)	0.78
Tenderness of Enthesis N (%)	96 (58.8)	19 (61.2)	0.80

Radiological and biological characteristics of the population

The comparison of radiological and biological parameters showed a higher prevalence of coxitis in the early SpA group (p=0.008). The other parameters were not significantly different between the two groups (Table 2).

**Table 2:** Radiological and biological characteristics of the population MRI: magnetic resonance imaging; ESR: erythrocyte sedimentation rate; CRP: C-reactive protein; * p value ≤ 0.05

Parameters	Early-onset SpA (N=163)	Late-onset SpA (N=31)	p
Sacroiliitis on X-ray N(%)	128 (78.5)	27 (87.1)	0.85
Sacroiliitis on MRI	35(21.4)	4 (12.9)	0.75
Syndesmophytes N (%)	57 (34.9)	14 (45.1)	0.29
Enthesitis N (%)	96 (58.8)	19 (61.2)	0.80
Synovitis N (%)	111 (68.1)	23 (74.2)	0.50
Coxitis N(%)	73 (44.7)	6 (19.35)	0.008*
ESR (mm)	39.16	39.73	0.92
CRP (mg/l)	35.50	36.84	0.90
HLA-B27	16 (9.81)	2 (6.45)	0.30
Vitamin D (ng/ml)	21.58	19	0.69

Characteristics of disease activity in the population

Late-onset SpA had a higher count of tender and swollen joints while the disease activity, measured by BASDAI and ASDAS-CRP, was not different between the two groups (Table 3).

**Table 3:** Characteristics of disease activity in the population ASDAS-CRP: Ankylosing Spondylitis Disease Activity Score-C-reactive protein; BASDAI: Bath Ankylosing Spondylitis Disease Activity Index; BASFI: Bath Ankylosing Spondylitis Functional Index; VAS-F: Visual Analogue Scale-Fatigue; * p value ≤ 0.05

Parameters	Early-onset SpA (N=163)	Late-onset SpA (N=31)	p
Tender joint count (Mean)	4.45	8.18	0.01*
Swollen joint count (Mean)	1.55	3.95	0.02*
ASDAS CRP (Mean)	3.24	3	0.86
BASDAI (Mean)	4.70	5.09	0.56
BASFI (Mean)	5.09	5.20	0.91
VAS-F (0-10) (Mean)	6.23	6.33	0.88

Comorbidities and extra-articular involvement

The prevalence of depression, fibromyalgia, and hypercholesterolemia was higher in patients with late-onset disease (Table 4).

**Table 4:** Comorbidities and extra-articular involvement IBD: inflammatory bowel disease; * p value ≤ 0.05

Parameters	Early-onset SpA (n=163)	Late-onset SpA (N=31)	p
Depression N (%)	1 (0.6)	4 (12.9)	0.00*
Diabetes N (%)	7 (4.3)	3 (9.7)	0.21
Fibromyalgia N (%)	1 (0.6)	3 (9.7)	0.001*
Latent tuberculosis N (%)	11 (6.7)	2(6.4)	0.90
Hypercholesterolemia N (%)	1 (0.6)	2 (6.4)	0.01*
Hypertriglyceridemia N (%)	1(0,6)	0 (0)	0.89
Osteoporosis N (%)	20 (12.26)	2 (6.4)	0.34
Cutaneus psoriasis N (%)	11 (6.7)	2 (6.4)	0.49
IBD N (%)	16 (9.8)	4 (12.9)	0.84
History of uveitis N (%)	22 (13.5)	5 (16.12)	0.69

Therapies in the population

The univariate study did not show significant differences in the use of NSAIDs, analgesics, corticosteroids, and csDMARDs (Table 5).

**Table 5:** Therapies in the population NSAIDs: non-steroidal anti-inflammatory drugs; csDMARDs: conventional synthetic disease-modifying antirheumatic drugs

Parameters	Early-onset SpA (N=163)	Late-onset SpA (N=31)	p
NSAIDs N (%)	75 (46)	8 (25)	0.08
Analgesics N (%)	16(9.8)	3(9.6)	0.54
csDMARDs N (%)	90 (55.21)	17 (54.83)	0.66
Corticosteroids N (%)	34 (20.8)	9(29)	0.40

Logistic regression

Late-onset SpA was associated with increased tender and swollen joint counts, depression, fibromyalgia, and a lower frequency of coxitis (Table 6).

**Table 6:** Logistic regression results * p value ≤ 0.05

Parameters	IC 95%	OR	p
Swollen joint count	0.85-0.99	0.92	0.03*
Tender joint count	0.88-0.98	0.93	0.01*
Coxitis	0.11-0.75	0.29	0.01*
Depression	0.04-0.38	0.19	0.005*
Fibromyalgia	1.74-17.85	1.75	0.01*
Hypercholesterolemia	0.98-127.25	1.65	0.06

## Discussion

The various subgroups of SpA can start after the age of 45, including ankylosing spondylitis, psoriatic arthritis, SpA with IBD, reactive arthritis, and undifferentiated SpA. The frequency of late-onset SpA remains poorly known and underestimated [3,4]. Consequently, late-onset SpA can present a diagnostic and therapeutic challenge. Their clinical presentation is less typical with a higher frequency of peripheral involvement and a lower frequency of axial involvement. Furthermore, late-onset SpA is not a diagnostic priority in the elderly compared to metabolic, infectious, and paraneoplastic diseases [7-9]. Late-onset SpA is characterized by a lower incidence of the HLA-B27 which could explain the delayed onset of symptoms [7,10,11]. However, studies by Tourisseau et al. and Weber et al. found a 70% frequency of the HLA-B27 antigen in late-onset SpA [12,13]. Radiography and magnetic resonance imaging (MRI) of the sacroiliac and spine are difficult to interpret in these patients, given the frequency of degenerative abnormalities. No study has evaluated the contribution of imaging in the diagnosis of late-onset SpA [3,7,13]. The assessment of disease activity may be influenced by satellite aspects of aging, including longer morning stiffness, sarcopenia, osteoarthritis, and comorbidities. Thus, the BASDAI, ASDAS, BASFI, and Bath Ankylosing Spondylitis Metrology Index (BASMI) scores may be overestimated in these patients, which implies the implementation of more adapted tools for these patients or the redefinition of activity thresholds for late-onset forms [7,13-15]. The ASAS 2009 criteria for axial SpA and 2011 criteria for peripheral SpA have been validated on patients under 45 years of age, so they are not suitable to use in subjects who develop the disease after this age. Nonetheless, one study suggested that these criteria could be employed when diagnosing patients who develop the disease after 45 years [16,17]. From a therapeutic perspective, the prevalence of comorbidities in older patients, such as heart disease, hypertension, stroke, renal failure, and infections, restricts the use of effective drugs like NSAIDs and biotherapies and requires a vigilant monitoring system and dose reduction [16,18]. Furthermore, the therapeutic sensitivity, particularly to NSAIDs, could be lower [12]. This study revealed a prevalence of late-onset forms of 16%. These patients had a higher prevalence of peripheral joint involvement, depression, and fibromyalgia, but a lower frequency of coxitis. Studies conducted in this area have shown different outcomes depending on the population. A study on a Japanese population of 114 patients illustrated that SpA with onset after the age of 50 years has a low frequency of IBD, a low prevalence of the HLA-B27 antigen, a high frequency of dactylitis, and a higher Doppler activity in peripheral joints [19]. Data from the Brazilian Registry on a population of 1424 patients showed that late-onset SPA was mostly female, with more dactylitis, peripheral arthritis, nail involvement, and psoriasis, but had a lower frequency of inflammatory low back pain, alternating buttock pain, radiographic sacroiliitis, coxitis, family history of SpA, HLA B27 antigen, and uveitis [20]. A study from the Spanish registry (REGISPONSER) indicated that 3.5% of 1257 patients had late-onset SpA after 50 years of age, with more cervical involvement and peripheral arthritis of the upper and lower limbs [21]. A study of 228 patients found an association between late-onset SpA and the involvement of the cervical spine and peripheral joints [22]. A study conducted on 76 patients with psoriatic arthritis revealed that late-onset forms were more inflammatory with an increase in ESR and CRP, more synovitis, and edema of the hands and/or feet. It should be noted, however, that psoriatic arthritis is not rare in the elderly compared to other rheumatic diseases, but several studies have reported a different phenotype of the disease as is also the case for cutaneous psoriasis before and after 40 years of age [23-25]. The differences in the results of the studies are explained by the variable age limits for defining late-onset forms, the variable inclusion and exclusion criteria between the studies, and the particular characteristics of each population, particularly the prevalence of the HLA-B27 antigen.

This study has several limitations, notably its small sample size and retrospective nature. Further prospective studies on a larger sample will be necessary to confirm the results of our study and to identify other particularities of late-onset SpA.

## Conclusions

Late-onset SpA is a distinct phenotype compared to early-onset forms. This study found that this phenotype was associated with a high number of tender and swollen joints, depression, fibromyalgia, and a lower frequency of coxitis. With increased life expectancy, the prevalence of late-onset SpA is expected to increase. Therefore, it is essential to define and validate diagnostic criteria for individuals over 45 years old and to include them in epidemiological studies and clinical trials to confirm diagnostic and therapeutic strategies specific to this population.
