# Correction: Characteristics of Late-Onset Spondyloarthritis: Data from the Moroccan Registry of Biological Therapies in Rheumatic Diseases

**DOI:** 10.7759/cureus.c121

**Published:** 2023-05-31

**Authors:** Ahmed Mougui, Zineb Baba, Ihsane Hmamouchi, Redouane Abouqal, Ahmed Bezza, Fadoua Allali, Rachid Bahiri, Imad Ghozlani, Hasna Hassikou, Linda Ichchou, Saadia Janani, Taoufik Harzy, Redouane Niamane, Abdellah El Maghraoui, Imane El Bouchti

**Affiliations:** 1 Rheumatology, EzarriHospital, Faculty of Medicine and Pharmacy of Marrakech, Mohammed VI University Hospital, Marrakech, MAR; 2 Rheumatology, Arrazi Hospital, Faculty of Medicine and Pharmacy of Marrakech, Mohammed VI University Hospital, Marrakech, MAR; 3 Laboratory of Biostatistics, Clinical Research and Epidemiology, Mohammed V University, Rabat, MAR; 4 Laboratory of Biostatistics, Clinical Research and Epidemiology., Mohammed V University, Rabat, MAR; 5 Rheumatology, Military Hospital Mohammed V, Ibn Sina University Hospital, Rabat, MAR; 6 Rheumatology B, El Ayachi Hospital, Ibn Sina University Hospital, University Mohamed V, Rabat, MAR; 7 Rheumatology A, El Ayachi Hospital, Ibn Sina University Hospital, University Mohamed V, Rabat, MAR; 8 Rheumatology, Faculty of Medicine and Pharmacy, University Ibn Zohr, Agadir, MAR; 9 Rheumatology, Military Hospital Moulay Ismail, Hassan II University Hospital, Meknes, MAR; 10 Rheumatology, Mohammed VI University Hospital, Mohammed I University, Oujda, MAR; 11 Rheumatology, Ibn Rochd University Hospital, Casablanca, Morocco, Casablanca, MAR; 12 Rheumatology, Hassan II University Hospital, Fes, MAR; 13 Rheumatology, Military Hospital Avicenne, Mohammed VI University Hospital, Marrakech, MAR; 14 Rheumatology, Private Medical Office, Rabat, MAR; 15 Rheumatology, Ezarri Hospital, Faculty of Medicine and Pharmacy of Marrakech, Mohammed VI University Hospital, Marrakech, MAR

This article has been corrected at the request of the authors to edit data that had typographical errors in the initial published version:

In Table 6, column 3, rows 3-6 have been updated from:

0.93

0.97

0.75

0.65

To: 

0.29

0.19

1.75

1.65

The corresponding data in the abstract has also been updated accordingly. The authors deeply regret that these errors were not identified and addressed prior to publication.

 

